# miRNAture—Computational Detection of microRNA Candidates

**DOI:** 10.3390/genes12030348

**Published:** 2021-02-27

**Authors:** Cristian A. Velandia-Huerto, Jörg Fallmann, Peter F. Stadler

**Affiliations:** 1 Bioinformatics Group, Department of Computer Science, and Interdisciplinary Center for Bioinformatics, Leipzig University, D-04107 Leipzig, Germany; 2 Max Planck Institute for Mathematics in the Sciences, D-04103 Leipzig, Germany; 3 Institute for Theoretical Chemistry, University of Vienna, A-1090 Wien, Austria; 4 Facultad de Ciencias, Universidad National de Colombia, CO-111321 Bogotá, Colombia; 5 Santa Fe Insitute, Santa Fe, NM 87501, USA

**Keywords:** MicroRNA, homology search, RNA secondary structure, multiple sequence alignment, consensus structure, repetitive element

## Abstract

Homology-based annotation of short RNAs, including microRNAs, is a difficult problem because their inherently small size limits the available information. Highly sensitive methods, including parameter optimized blast, nhmmer, or cmsearch runs designed to increase sensitivity inevitable lead to large numbers of false positives, which can be detected only by detailed analysis of specific features typical for a RNA family and/or the analysis of conservation patterns in structure-annotated multiple sequence alignments. The miRNAture pipeline implements a workflow specific to animal microRNAs that automatizes homology search and validation steps. The miRNAture pipeline yields very good results for a large number of “typical” miRBase families. However, it also highlights difficulties with atypical cases, in particular microRNAs deriving from repetitive elements and microRNAs with unusual, branched precursor structures and atypical locations of the mature product, which require specific curation by domain experts.

## 1. Introduction

MicroRNAs (miRNAs) emerged 20 years ago as an important class of endogenous small non-coding RNAs with key functions in post-transcriptional gene silencing affecting a sizeable fraction of protein-coding mRNAs [1]. Originally described in animals and plants, miRNAs meanwhile have also been reported in several major groups of unicellular eukaryotes [2]. Their molecular function builds upon the presence of the evolutionarily even older RNA interference pathways that inactivate double-stranded RNA [3]. The most recent release of miRBAse (22.1) [4] lists 1984 miRNA precursor hairpins, and a recent extrapolation estimates about 2300 mature microRNAs for human [5]. These numbers are much larger than those reported for other mammals, suggesting that our knowledge of the miRNA repertoire of animal genomes is still far from complete. On the other hand, reference [6] counts only 519 “confidently identified canonical miRNA genes”, see also [7]. The discrepancy derives both from the level of experimental evidence required to confidently identify a ncRNA gene and from the *definition* of what constitutes a canonical miRNA, as opposed to a member of a wider class of small RNAs associated with the RNA-interference pathways, see, for example, [8,9,10].

Despite their small size of only about 80–100 nt, miRNA precursors are readily detected by sequence comparison due to the often extreme level of sequence conservation [11]. Homology-based methods are thus at least in principle capable of assessing the phylogenetic distribution of individual miRNA families [12]. Since miRNA families are rarely lost, miRNAs have been proposed as a powerful phylogenetic marker in animal phylogeny [13,14,15]. A detailed, quantitative evaluation of miRNA evolution is not at all straightforward, however. Several recent studies have pointed out the problems of simplistic approaches—on the one hand, there is an ongoing discussion about how miRNAs should be distinguished from other, functionally similar classes of small RNAs. We focus here on *canonical miRNAs* that are processed by Drosha and Dicer and thus share several common features [16]: (a) evidence for the expression of both the mature miR and its miR* from the opposite side of the precursor hairpin; (b) a sequence distinct from the processing products of other structured RNAs such as rRNAs, tRNAs, snRNAs, or snoRNAs; (c) a sequence that is non-repetitive in the genome of origin; and (d) consistent 5’ processing of both the miR and the miR* sequence. On the other hand, utilization of simple, blast-based homology searches alone tend to produce false positives that require extensive curation, which largely relies on the properties expected for a miRNA [17]. The features of canonical miRNAs can be translated in computational rules for the evaluation and editing of structure-annotated alignments of miRNA families that can be utilized to determine whether a candidate sequence fits to a known miRNA family or whether it constitutes a false-positive candidate [18,19].

Homology search for pre-miRNAs, as for other families of structured RNAs benefits substantially from including information on the consensus structure. This is achieved naturally using covariance models (CMs) [20,21] as implemented in Infernal [22]. The accuracy and sensitivity of Infernal, however, depends critically on the quality of the sequence alignment and the annotated consensus structure that is used to build the CM. Although the Rfam database [23] already provides curated alignments and secondary structures for many miRNA families, it is far from complete. A potentially even more serious problem with the Rfam miRNA alignments is that family-membership is much more loosely defined than miRNA families in miRBase [4], where stringent homology criteria are used. Some of the Rfam miRNA alignments are thus composites of miRBase-families. miRBase, on the other hand, contains more sequences, but does not provide curated alignments and consensus structures. The precursor sequences available for a given family often have inconsistently defined ends, and the alignments for different families tend to differ in which species they include. Recent work also identified a moderate number of erroneous entries [19], see also [24].

These observations call for an integrated workflow to perform homology search and to evaluate the search results in a consistent manner. In this contribution we improve on ideas from MIRfix [19] and integrate it with homology search. miRNAture is specifically designed to identify and annotate metazoan miRNAs in a homology-based setting. The miRNAture pipeline thus is complementary to the tools and pipelines that extract miRNA candidates from small RNA-seq data, such as mirDeep2 [25], miRTRAP [26], or miRRim2 [27]. Homology search for small, structured RNAs is inherently limited by the small sequence length and the comparably rapid evolution of (at least) parts of the molecule. The trade-off between sensitivity and specificity, which is unavoidable for automatized methods, thus sets a detection limit that infringes on systematic studies of ncRNA evolution also in practice [28]. The balance between sensitivity and specificity of generic methods (including blastn [29], nhmmer [30] and cmsearch [22]) can be tipped in favor of sensitivity if the queries are restricted to certain RNA classes for which highly specific post-filters can be constructed. An example is tRNAscan-SE [31], which achieves near perfect recovery of canonical tRNAs at the expense of being applicable to tRNAs only. The miRNAture pipeline follows the same philosophy for canonical miRNAs—the initial acquisition of candidates using generic homology search tools operates at increased sensitivity and returns large numbers of false-positives candidates, which are then weeded out by very stringent, miRNA-specific filters.

This contribution is organized as follows—the strategy and organization of the miRNAture pipeline is outlined in Section 2.1, while the details of the individual components of the workflow are described in the Methods Section 4. We then consider two show-case applications. In Section 2.2 we re-analyse the *let-7* family across vertebrates, before testing the pipeline on the human miRNA complement after excluding all human sequences from the queries. Both show-case applications are concerned with data that have been analysed extensively in the past and thus allow us to point out both strengths and weaknesses of miRNAture and the published miRNA annotations. The source code of miRNAture, together with the technical description are available at: https://github.com/Bierinformatik/miRNAture (accessed on 26 February 2021).

## 2. Results

### 2.1. Architecture of miRNAture

**Figure 1:**
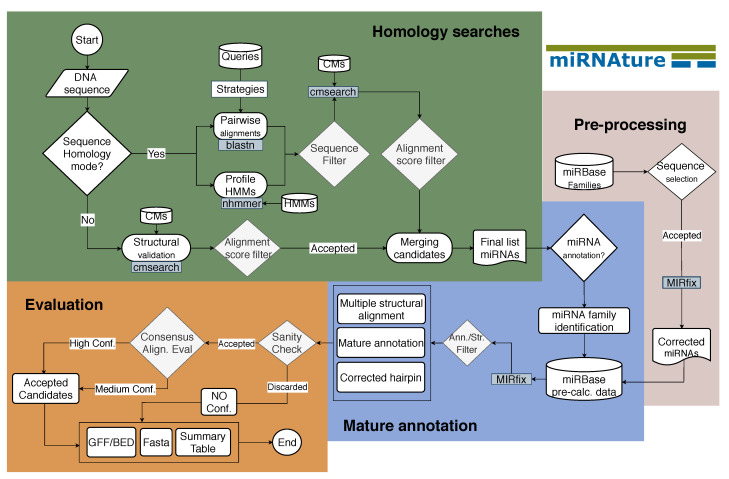
Workflow of miRNAture. The starting point is the provided set of target sequence by the user, which is first analyzed in *Homology search* mode to detect miRNA candidates. Specifically, two strategies are available: sequence homology and structural validation. The first one using pairwise alignments, performed with blastn, or hidden markov models (HMMs) using nhmmer. The second one is based on the use of covariance models (CMs). Each of the described stages has their own filters, accepted candidates being submitted to the next stage while discarded candidates are reported separately for later manual inspection. A merging step produces a final list of homology candidates. After that, *Mature annotation* stage runs on these input sequences and performs a correction of the positioning of mature sequences on the hairpin, generating a correctly anchored family-specific-multiple secondary structure alignment (calculated by MIRfix [19]). The *Evaluation* stage starts with a sanity check that reviews the mature annotation and performs a comparison of conserved secondary structures with and without the newly annotated candidates. Based on this classification the candidates will be labeled as accepted or discarded. Sequence length and minimum free energy (MFE) cutoffs are used for further filtering. A final set of candidates is reported in BED/GFF3 annotation formats and FASTA files. A summary file provides overall information about the miRNA candidates and families and contains additional candidates which failed or have not been considered for evaluation due to cutoffs for manual inspection.

The miRNAture pipeline is composed of three modules: (1) *Homology* search operating on miRNA precursors; (2) prediction of the positioning of mature miRNAs within the precursor (*Mature annotation*); and (3) an *Evaluation* scheme designed to identify false positive miRNA annotations. The pipeline is distributed with pre-computed CMs for the miRNAs in Rfam v.14.4 [23], which are used as default for annotation of a target sequence or genome. Users can also add their own CMs, and/or a query sequence that will subsequently be annotated. It is also possible to use a combination of built-in and user supplied CMs. miRNAture produces annotation files in GFF3/BED format and FASTA files for validated candidates as well as summary reports that highlight possibly problematic cases, tagging these for manual inspection. The architecture of miRNAture is summarized in Figure 1.

In the initial step, miRNAture can use either individual miRNA sequences or pre-computed/user provided CMs. In sequence mode, miRNA-specific strategies based on blastn [32] are employed. These are discussed in [33,34], full details on the parameter choices are given in Supplementary Table S1. In the following filtering step, overlapping blastn hits are aggregated into *extended regions* as described in Supplementary Section 1 and Supplementary Figure S1. Alternatively, HMMs of miRNA families pre-computed from Rfam v.14.3 stockholm alignments, or user defined ones, for example, inferred from miRBase [4] can be compared against the target genome using nhmmer [30] to determine initial candidate homologs. If CMs for the query families are available, the initial datasets are evaluated w.r.t. structural alignments using cmsearch [22]. miRNAture also offers the option to search the target genome with user-defined CMs. These can be obtained, for example, from alignments of *miRBase* sequences, directly from *Rfam*, or from the user’s own alignments.

Independent of the strategy chosen for the initial step, the candidate sequences are then filtered based on specific threshold values: *E-value*, coverage and if available, bitscore (using family CM threshold value defined by Rfam as *gathering cutoff* (https://docs.rfam.org/en/latest/choosing-gathering-threshold.html (accessed on 1 February 2021)). Then miRNAture disambiguates the reading directions in case overlapping candidate loci at both strands have been detected. To this end, quality measures provided by *Infernal* (*bitscore*, *E-value* and *coverage*) are used. The final candidate lists are merged. For each candidate, coordinates and the supporting initial hits are reported. This three-fold search might seem redundant at first glance as, not surprisingly, there is a large overlap between the search results. However, as can be seen in Supplementary Figure S2, some miRNAs are found only with one but not the other search method. The increased sensitivity, thus, justifies the extra effort, in particular when the aim is a comprehensive, high-quality annotation.

In the next step, miRNAture attempts to identify the location of the mature miR and miR* within the preliminary precursor sequences. To this end, we adapted MIRfix [19] and released version 2.0.0 (https://github.com/Bierinformatik/MIRfix/releases/tag/v2.0.0, accessed on 26 February 2021). Mature miR/miR* sequences were obtained from miRBase. For each family, the result of this step is an alignment of corrected and trimmed miRNA precursor sequences annotated with the placement of the mature sequences and finally a structure-annotated sequence alignment. Corrected alignments were pre-calculated for miRBase families only and are available together with the corresponding CMs here: http://www.bioinf.uni-leipzig.de/publications/supplements/21-001 (accessed on 26 February 2021).

In the final stage, the corrected, structure-annotated alignments are used to evaluate homology search results. Since miRNA hairpins form extremely stable RNA secondary structures [35], we can use direct structure prediction and comparison with the consensus structure of the family to measure how well new candidates structurally conform to a given RNA family. Together with sequence length, folding energy and sequence blocks conforming to the mature miRs, this provides a reliable filtering procedure, summarized in Table 4 in the Methods Section 4.

Testing the performance of miRNAture in terms of measures like sensitivity or precision requires a dataset with a reliable ground truth of positive and negative instances. Such a dataset, however, is currently not available for miRNAs despite extensive efforts of the curators of miRBase [4]. On the one hand, only positive data, that is, miRNAs with sufficient support, are reported making it impossible to quantify specificity. Spurious annotations [17] and unclear boundaries of what exactly constitutes a miRNA [10], on the other hand, compromise the quantification of specificity. An alternative strategy to evaluate the performance is to use simulated data. This, however, requires an *independent* method to generate artificial data, in our case alignments of miRNA families. To our knowledge, no such tool is available. We shall return to this issue in the discussion section. We will, however, use a simple simulation to asses some properties of miRNAture’s filters. Instead of a quantitative evaluation of miRNAture, we therefore consider two scenarios in which a semblance of the ground truth is known from extensive manual curation—the history of the *let-7* family and the human miRNA complement. In both cases the discussion will focus on the differences between current annotation on the findings of miRNAture.

The miRNAture pipeline can be downloaded from https://github.com/Bierinformatik/miRNAture (accessed on 26 February 2021). It is provided as Conda package for installation, which resolves all dependencies and includes a detailed user manual, a tutorial and extensive example data.

### 2.2. Annotation of let-7 on Chordate Genomes

The *let-7* family [36] is one of the most conserved families through metazoan species [12,13,37,38]. It is also one of the largest miRNA families in vertebrates with paralogs appearing both in tightly linked clusters and distributed across several chromosomes [12,39]. Since the evolution of the *let-7* family was studied extensively in the past [39,40,41,42] it provides probably the best available reference data set. In order to test consistency of the results obtainable with miRNAture, we performed re-annotation experiments with several primate genomes, the mouse genome and the Pacific transparent sea squirt, *Ciona savignyi*, as targets. In each case, all miRNAs annotated for the target genome were removed from the alignments and CMs of the query, see Section 4.3 for methodological details. To consider missing annotations in miRBase, we intersect miRNAture derived *let-7* loci also with the manual annotation of Hertel et al. [40]. The latter, together with miRBase annotation (MIPF0000002), are considered our *gold standard* annotation. Table 1 summarizes the results for the *homology* stage and the *final* stage of miRNAture, respectively. In summary ≥91% of all annotated *let-7* loci in all species were recovered by miRNAture, while in all cases, except the solitary tunicate, one of the annotated loci was not identified. Furthermore, between 1 and 11 additional loci per genome are considered valid, novel *let-7* candidates. In the following paragraphs we discuss these results in detail.

The missing candidates correspond to locus *K-let-7* in all primates, in the nomenclature of Hertel et al. [40], which considers homology of paralogs based on synteny. Hertel et al. [40] reported those loci as primate-specific novel candidates based on homology. However, the consensus structure generated from a multiple alignment with annotated *K-let-7* sequences shows a multi-loop structure where the typical miRNA hairpin is expected, while *G-let-7-1* was detected in the mouse genome considering only the homology stage, but discarded by structural filters in the evaluation stage for similar reasons. See Supplementary Figure S3 and Supplementary Section 3 for details.

Since the *homology* stage of miRNAture is optimized for sensitivity and only *let-7* was used a query, *bona fide* miRNAs that share some similarity with *let-7* are expected to have passed filters. The additional *let-7* loci found by miRNAture were therefore compared to the annotation of other miRNA families. We indeed found overlaps with miRBase annotation for the human specific *hsa-mir-4699* (MI0017332), and the families *mir-3596* (MIPF0001194), and *mir-625* (MIPF0000534). A *mir-3596* was annotated in *Rattus norvegicus* and identified by miRNAture also in mouse. The *mir-625* family was know in human and macaque only. These cases account for a third of the additional matches. Almost all of the remaining loci overlap with regions annotated as repeats. Only three loci (human: chr1:16082685-16082783:+, chimpanzee: AACZ04010697:5895-5965:-, and *C. savignyi*: reftig_41:1114844-1114937,+) do not overlap with available annotation. The similarity of *mir-625* and *let-7* was noted before e.g., by Rfam, which includes *mir-625* in their *let-7* miRNA precursor family RF00027. In [41], *mir-3596* is treated as a member of the *let-7* family, highlighting that miRNAture presumably classified them correctly as novel candidates.

#### 2.2.1. Simulation of Artificial *let-7* Instances

To check the behavior of miRNAture in the presence of large sequence divergence we artificially mutated two of the human *let-7* genes (chr21:16539829-16539913:+ and chr3:52268269-52268368:−) with increasing number of mismatches. For up to 10 point mutations, the loci were recovered at the homology stage, 4 candidates passed the homology filters and 2, overlapping the original loci, also survived the structural filters. At higher artificial mutation rates none of the initial candidates satisfied the structural constraints. As expected, at even higher mutation rates eventually also the initial homology search fails.

### 2.3. Annotation of Human *Bona Fide* miRNAs

A typical use case for miRNAture is the annotation of a genome of interest with a set of available miRNA family CMs. To simulate such a use case and simultaneously further benchmark miRNAture, we used a set of 350 miRNA families with a human entry in miRBase v.22 to construct query alignments from which all human sequences were removed, see Supplementary Section 2 for details.

We find that, at the *homology* stage, miRNAture detected miRNA candidates for all but a single family. Considering the annotation of mature sequences and curation at structural level with MIRfix [19] in the validation stage, candidates for 323 families (92.23% of initial miRNA CMs) were retained. For 27 families candidates were found on homology level, but later discarded based on the evaluation of structure and localization of mature miRNA regions within the hairpin.

In order to better understand the performance of miRNAture at the *homology* stage we distinguish families where all candidates overlap exactly with annotation (337 families, 96.3%) and those where a part of the candidates overlap (12 families). The only family that was not recovered at all is *mir-297* (MIPF0000204), with 100 initial candidates, of which 69 passed the filtering steps. However, none of them matched the annotated loci. In mouse, six precursor loci have read support at mature sequence loci of which 4 are annotated by miRBase as *high confidence* miRNAs. Additional homologs at a single locus without read support are annotated in rat and some primate genomes: *Macaca mulatta*, *H. sapiens* (very weak read support) and *P. troglodytes*. Input for miRNAture was a CM model built from mouse validated sequences, which in comparison to the known human locus contain 20% more nucleotides and 10% additional consensus positions.

Of the 323 families left after the homology stage, most show a perfect match with current annotation for all accepted candidates (87.9%), see Table 2. The final output of miRNAture comprises 284 (81.1%) families with perfect overlaps with the current annotation. Another 28 families show partial matches. Among the remaining 38 families, there are 27 for which no candidate passed the filtering steps. The other 11 families contain additional candidates, but are disjoint from the current annotation (do not show overlap). Table 2 summarizes the statistics.

The 11 families with candidates disjoint from human annotation are *mir-1233*, *mir-1291*, *mir-1306*, *mir-140*, *mir-6127*, *mir-645*, *mir-652*, *mir-764*, *mir-873*, *mir-877* and of course *mir-297*. For annotated loci of these miRNAs that were not recovered at least one of the following statements are true: (a) There is no mature sequence alignment available that allows the correct annotation of detected candidates; this is in particular the case for species-specific families. (b) The location of the mature sequences was determined based on similarity to human loci alone, without additional information. With the artificial removal of the human data this information is unavailable in our benchmark. (c) The miRNAture pipeline favors the opposite strand. Details can be found in Section 2.3.4 and Supplementary Table S5.

For 27 families, all candidates where **Filtered** out even though they show a perfect match with the current annotation. These cases can be traced back to miRNAs which belong to either species specific families, thus lacking homology information, or have only been found in a small set of other species, consequently restricting the available dataset of mature loci, or folding into invalid secondary structure. In total, this lead to rejection of 33 loci, see Section 2.3.4 and Supplementary Table S6 for details.

In summary, the loci that miRNAture did not cover in the human genome fall into two broad classes: (1) members of repetitive families, for which we consider it uncertain whether they should be considered as canonical miRNAs; (2) precursors with deviant secondary structure or unusual placement of the mature sequences within the predicted secondary structure. These families deserve a closer look whether they are canonical miRNAs in the stringent sense used here. If so, they may prompt a future adjustment of the filtering criteria; (3) Families for which the query alignment and secondary structure contains an insufficient number of precursor sequences or contains undetected errors in alignment, positioning of the mature sequences, or consensus structure annotation. We regard them as border line cases that deserve further investigation into the underlying evidence, preferably from multiple species—a task that goes beyond the scope of this contribution.

#### 2.3.1. Additional Candidates

For 178 families (1366 loci) additional candidates were predicted. At the same time, 5 families (6 loci) were removed by filtering steps. A comparison with the current annotation shows that ∼69.0% of those *additional* loci overlap with one or more annotated element(s) (see Table 3). For 12 families we found candidates that overlap with annotation of other miRNA families (different), while for 73 families we find overlaps with repeat regions (repeat), and 31 which overlap with other annotation (other) including, for example, intronic or exonic regions of lncRNAs or coding genes. For the *mir-1233* family, for instance, 20 additional loci were reported. Almost all of them are located on chromosome 15 and overlap retained introns or lncRNAs derived of the palindromic GOLGA8 gene family, described as core duplicons dispersed along ∼14 kbp, associated to structural variants and genomic instability regions in general [43,44].

Twelve miRNA families show overlaps with other miRNAs that are annotated as human-specific, see Supplementary Table S7 for details. An exceptional case is *hsa-mir-499b* (MI0017396). The homology stage of miRNAture suggests that it belongs to the *mir-499* family, however, miRBase does not include it in this family. We argue that *hsa-mir-499b* is correctly annotated by miRNAture.

#### 2.3.2. Additional miRNAture Candidates without Annotation Overlaps

**Figure 2:**
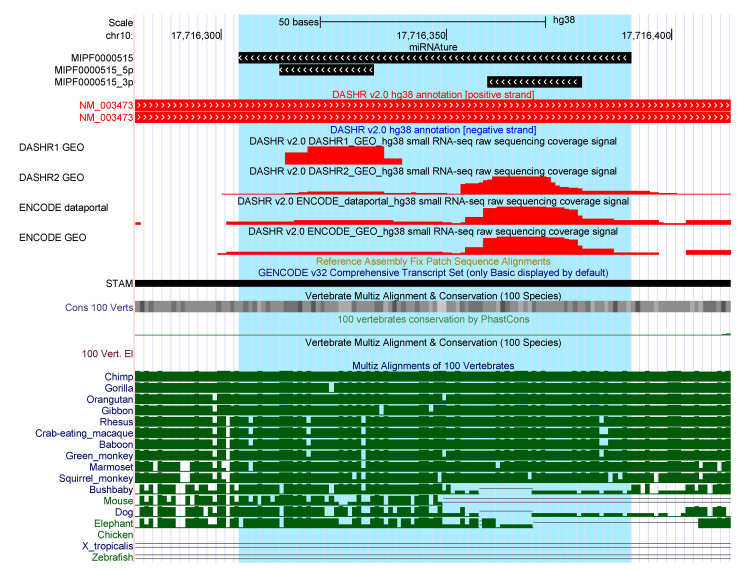
Crossed annotation and expression patterns overlapping in the same region where a loci of *mir-580* was detected by miRNAture. Red tracks correspond to sRNA-Seq mapped reads, reported from [45].

For 129 families (423 loci) miRNAture predicted candidates that do not overlap with any currently annotated genomic element on the same strand. The miRNA families *mir-544* (50), *mir-548* (42), *mir-1302* (27), *mir-1289* (21), *mir-649* (19), *mir-290* (17), and *mir-297* (11) account for nearly half of them. To further investigate those candidate loci, we intersected available annotation specifically at their ‘antisense’ strand and found 105 overlaps. Interestingly, more than half (53.9%) of those are found in overlap with repetitive elements, (24.82%) overlap with a miRNA annotated on the opposite strand while in 5.67 a coding gene and in 0.94% a lincRNA is annotated in antisense. Integration of expression patterns derived from a small RNA-Seq dataset from Kuksa et al. [45] revealed read support for ∼8% of these 105 candidates. As example, Figure 2 show a *mir-580* precursor. It was predicted in antisense to the 3’UTR of the protein coding gene *STAM*, a locus well conserved among primates. More examples are shown in Supplementary Figures S4, S5 and S7.

Many of the additional loci overlap specific repeat families, see Supplementary Table S4. All additional *mir-544* loci overlap with the DNA transposon MER (*medium reiterated frequency repeat*). It is interesting to note that the annotated *mir-544* loci are located in the DLK1-DIO3 imprinted region [46]. Similarly, 42 *mir-548* loci overlap with Tc1/Mariner. An alignment of the *mir-548* predictions is shown in Supplementary Figure S6. The extensive, repeat-like *mir-548* family has received detailed attention in the past [47,48], highlighting its atypical features deriving from Made1 elements, a class of inverted-repeat transposable elements (MITEs). A large number of *mir-548* loci have been reported to match Made1 elements in both reading directions [47]. The family also features an atypically large divergence among their mature sequences. Some paralogs share the same locus on different strands and generate miRNA:miRNA* duplexes lacking the otherwise typical hairpin loop region [48].

Furthermore, 12 loci from 11 families passed the validation stage, but the predicted position in the human genome is not overlapping with annotation (see Supplementary Table S5). A better fit of the mature sequences was found for the opposite strand in 3 cases: *mir-764*, *mir-140* and *mir-1306*.

#### 2.3.3. Strand-Mismatch Candidates

**Figure 3:**
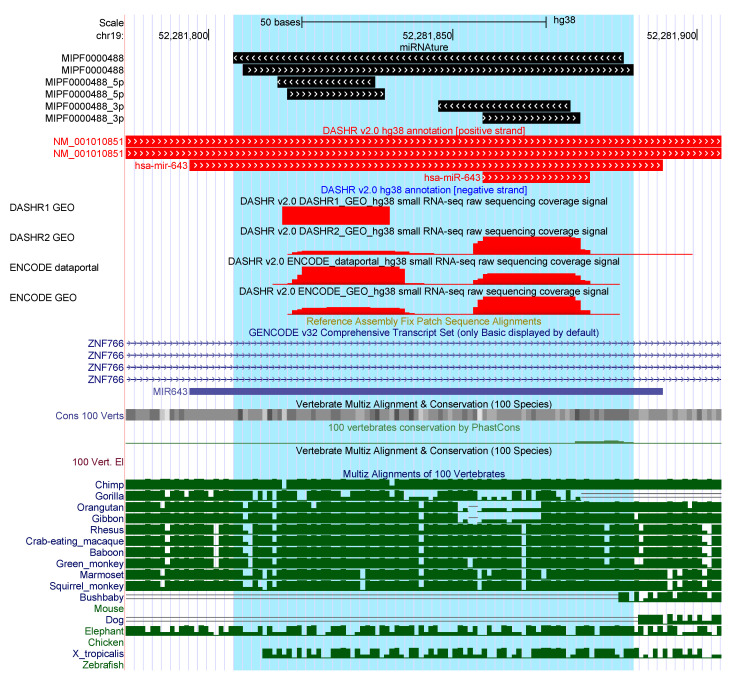
Example of overlappings with current miRNA annotation in human genome. Annotated *mir-643* loci were detected by miRNAture on the same strand and additionally an opposite locus from the same family was detected. Supporting expression patterns were detected by both, 5’ and 3’ miR; however, currently only on the 5’ miR is annotated.

For six loci that pass all evaluation steps of miRNAture, the strand may be mis-annotated, Supplementary Table S3. In each case, valid homology regions were detected on both strands, the opposite strand was preferred by miRNAture based on the prediction of unusual positions of the mature sequences in relation to the secondary structure for the other strand. Examples are overlap of the mature sequence and the hairpin loop, or a multi-loop structure, see Figure 3. Predictions that match better to the opposite strand than the annotated locus were found for the following families: *mir-101*, *mir-103* (2 loci), *mir-122*, *mir-1245*, *mir-290*, *mir-451*, *mir-4536*, *mir-515*, and *mir-548*. Difficulties with the *mir-451* family are not unexpected due to its atypical biogenesis and a dominant mature product deriving from the loop region [49].

#### 2.3.4. Missing Candidates

At the homology stage of miRNAture, 90 annotated miRNA loci from 13 families were not recovered. The majority of which were not reported due to the large number of detected homolog loci since miRNAture limits candidate lists to the 100 loci with the best bit-scores. This cut-off is intended to exclude candidates associated with highly repetitive sequences for which different, synteny-aware methods have to be used, see, for example, reference [50]. Candidates not passing this cut-off are flagged as *potential* candidates for later inspection by the user. 68 missing candidates can be explained in this manner. For example, *mir-548* represents a highly repetitive miRNA (with 74 annotated loci in miRBase). miRNAture detected in total 6626 candidates by homology searches, highlighting the need for stringent cut-offs. From them, the best 100 bit-score candidates were subject to mature annotation and compared to the annotation, 63 were classified as *potential* and another 2 were predicted on the opposite strand. Among the remaining 22 of the 90 *missing* loci belonging to non-repetitive families are three predictions on the opposite strand (*hsa-mir-103b-1* MI0007261, *hsa-mir-103b-2* MI0007262 and *hsa-mir-371b* MI0017393) and miRNAture rejected a total of 19, including five *mir-548* paralogs, four *mir-378* sequences, and three *mir-506* loci, while the remaining 7 undetected loci were not recognized by the corresponding HMM or CM miRNA family models.

In the final result, that is, after the validation stage, 161 loci from 66 families are classified as *missing*, see Table 2. Of these, 45 loci from 38 families were rejected, either at the validation stage (27 families) or did not shown genomic loci overlap (11 families) with annotation. For the first group comprising 33 loci, only a limited set of annotated mature sequences was available, and predicted mature sequences were incorrectly placed, so that the loci were eventually rejected. The *mir-550* family (MIPF0000334), for example, has five loci annotated in human. These were rejected by miRNAture because the mouse and chimpanzee loci retained in the input did no pass the secondary structure filters. Similarly, all three *mir-1184* loci predicted at the homology stage were rejected because of the atypical secondary structure of the only remaining input sequences (from chimpanzee), see Supplementary Table S6 for further details.

Another source of *missing* candidates are the 28 families that matched the current annotation only partially, accounting for 116 loci. These include the ten loci assigned to opposite strand and the 66 highly repetitive loci tagged as *potential* that have been discussed above. Of the remaining 40 loci, 19 were not detected by homology and 21 were rejected by miRNAture at the evaluation stages. Here, the CM constructed from miRBase data after removing the human sequences did not match the annotated human loci. For example, *hsa-let-7g* was rejected at the validation stage because the common *let-7* CM identified a sequence that was shifted relative to the paralog-specific results of Section 2.2, resulting in a less stable, shifted MFE structure. While it perfectly matched the mature sequence platypus *oan-let-7g-5p*, human mature sequences overlap the hairpin region, explaining the rejection.

## 3. Discussion

The miRNAture pipeline is based on the observation that efficient homology search requires the interplay of fast and ideally loss-less identification of candidate loci in the genome of interest, and subsequent filtering to remove the false-positives. Since it is not difficult to increase the sensitivity of initial search (by simply lowering cut-off values), better and in particular more complete results can be achieved by developing more efficient filters. This is not a new principle, of course. HMMs improve over single-sequence blast queries by including patterns of sequence conservation, and covariance models provide another jump in accuracy by incorporating the conservation on secondary structure level. The trade-off, however, is the need for more and more information on the query side. While blast requires only a single sequence, nhmmer requires a multiple sequence alignment to derive the HMM model, and the CMs used by cmsearch need a consensus structure in addition to the sequence alignment. CMs thus are helpful only if the RNA family has an evolutionary well-conserved secondary structure.

miRNAture increases the achievable sensitivity by further restricting the scope of queries—its filters are highly specific for *canonical microRNAs*, i.e., those that share all the typical features of miRNA precursors, in particular a secondary structure that resembles a nearly symmetric stem-loop and a sequence conservation pattern governed by the location of the mature products on both sides of the stem region. Therefore, miRNAture tends to reject members of atypical families such as those associated with repetitive elements. The main use of miRNAture is to reliably process the typical cases and to limit the need for extensive manual analysis to miRNAs and miRNA-like ncRNAs with atypical features.

The integration of the mature sequences and the evaluation of folding energies reaches beyond the information captured by HMMs and even CMs. This yields more stringent filters that make it feasible to increase the sensitivity of the initial homology search. The cost incurred for this advantage are the restriction of miRNAture to canonical microRNAs. While general approaches can be extended to other classes of RNAs, such as box C/D snoRNAs or box H/ACA snoRNAs, class-specific filters need to be developed and tested. This requires extensive domain knowledge and thus makes it difficult to extend the strategy to poorly understood ncRNAs.

The miRNAture pipeline is designed specifically to facilitate homology search for canonical miRNAs. The most obvious use case is the annotation of all conserved miRNA families in one or more new genomes. Complementarily, studies into the evolution of specific miRNA families require that (i) distant homologs can be detected reliably and (ii) no spurious apparent homologs are included. Only then is it possible to pinpoint the evolutionary origin of a miRNA family [12,17,40]. Although miRNAture usefully assists both tasks, a number of issues remain that will require manual intervention and post-processing. Most importantly, the method relies on correct initial models for each microRNA. We recommend to use models that are specific to individual miRBase families, or—in the case of families with divergent paralogs—even paralog specific input alignments. While it is possible to use Rfam family models, these turned out to be too promiscuous in many cases, resulting in relatively large fractions of rejected candidates.

The study presented here also highlights the difficulty of benchmarking homology search tools for ncRNAs. The main reason is the lack of a gold standard of sufficient quality and coherence. Databases such as miRBase or Rfam by design contain entries that satisfy certain levels of evidence. These evidence criteria, however, imply massive ascertainment biases between organisms as a consequence to the large differences in the available empirical evidence. On the other hand, the definition of miRNAs as a class is fuzzy to certain extent as well, implying that not all database entries share all the features that are typical animal miRNAs. In miRNAture, very stringent quality criteria are implemented. While the evaluation against the human annotation shows that clear false positive calls are rare and largely confined to repeat-associated families, miRNAture fails on miRBase families with atypical features. The miRNAture pipeline also reports candidates of the homology stage that are later rejected by the automatic curation procedure to enable expert inspection. Such datasets are required to gather enough knowledge about miRNAs with atypical features.

In principle, it would be desirable to benchmark miRNAture and similar tools against simulated data with a guaranteed ground truth. The difficulty is that checking the sensitivity and specificity of the filters requires a way of simulating the evolution of artificial miRNAs that is independent of filter rules employed by miRNAture. It would be easy of course, to use miRNAture, that is, the MIRfix-based evaluation to model the selection pressures on miRNAs, but then our filters would be perfect by construction, and no information on the biological correctness of the filters could be gained. On the other hand, it is very simple to construct negative examples, since 10–20% of randomly placed point mutations is known to almost certainly destroy the secondary structure [51]. We have seen in Section 2.2.1 that this is indeed also the case in our setting and therefore resorted here to comparing miRNAture with the known, well-curated miRNA annotation of the human genome. Again a fully quantitative evaluation is difficult, because of the gray-zone between *bona fide* canonical miRNAs and other miRNA-like genes.

The strategy of miRNAture may serve as a blueprint for a new generation of homology search tools that rely on class-specific post filters. Here, we have manually constructed the homology and secondary structure filters, making use of explicit knowledge on structure and biogenesis of miRNAs. It seems tempting to use machine learning classifiers for miRNA gene detection (reviewed e.g., in [52]) as an alternative. However, the correlation between miRNAs used for training and their homologs are a concern that will require detailed evaluation before such a strategy can be employed safely. For the time being, explicitly constructed filters thus seem preferable.

## 4. Methods

### 4.1. Specific Filters on miRNAture

The sequence/structure filters can be grouped by type of evaluation: **Sequence homology**, **Alignment scores**, **Annotation/Structure**, and **Consensus secondary structure**. Table 4 summarizes how they are employed in the different modes of the miRNAture workflow.

Pairwise comparisons with user-defined query sequences in **Sequence homology** searches are evaluated in terms of E-values, coverage and length of resulting *high scoring pairs* (HSPs) as suggested in [34].

HMM comparisons are evaluated w.r.t. the default inclusion thresholds of the nhmmer models as suggested in the HMMer userguide (http://eddylab.org/software/hmmer/Userguide.pdf (accessed on 18 December 2020)). Direct comparisons to miRNA CMs make use of the parameters calculated by cmsearch: E-value, bitscore and coverage with respect to the length of the CM. An uniform bitscore cutoff of $log_{2}2N$ is used, where *N* denotes the genome size. If a *gathering cutoff* (ge) is available for a CM, for example, in Rfam models, miRNAture uses a threshold of 0.32ge to rescue candidates that potentially represent valid miRNAs. Structural parameters are evaluated with an updated version v2.0.0 of MIRfix [19] (https://github.com/Bierinformatik/MIRfix/releases/tag/v2.0.0 (accessed on 27 January 2021)).

The focus of this evaluation step is the correct annotation of mature sequences relative to the precursor. To this end, a precursor length of ≤200 nt and a secondary structure with a minimum free energy (MFE) ≤−10 is required. The additional *Evaluation* stage of miRNAture compares the *tree edit* distance [53] between the consensus structure dot-bracket string of the pre-defined structural alignments of the miRNA family and the re-computed alignment that includes the additional, new precursor sequence. The structural distance is used to determine the confidence level of the new candidate, which passes the validation stage: *High*: Valid consensus secondary structure and tree edit distance ≤7 to the consensus secondary structure of the initial family; *Medium*: if fails any of those. In case a large number of homologs is found, only a user-specified number of top candidates are processed as described. The remaining putative homologs are reported separately as putative matches.

### 4.2. Genomes

The following genome assemblies were retrieved from the Ensembl FTP site (Release 100): gorilla (*Gorilla gorilla*: gorGor4), chimpanzee (*Pan troglodytes*: Pan_tro_3.0), sumatran orangutan (*Pongo abelii*: PPYG2), human (*H. sapiens*: GRCh38.p13), mouse (*Mus musculus*: GRCm38.p6), and Pacific transparent sea squirt (*Ciona savignyi*: CSAV 2.0).

### 4.3. Curation of the let-7 Family

*Let-7 loci* from *G. gorilla*, *H. sapiens*, *P. abelii*, *P. troglodytes*, *M. musculus* and *C. savignyi* were retrieved from miRBase v.22 in FASTA and GFF3 format. In addition, the let-7 loci reported in [40] were retrieved and mapped to the genomes listed above. The union of the *let-7* loci from [40] and from miRBase were used as reference for evaluation.

CMs for *let-7* were retrieved from Rfam v.14.4 (RF00027), Hertel et al. [40] (17 models from the A, B, C, D, E, F, G, H, I, J, K and L paralogs), and Yazbeck et al. [19] curated (miRBase v.21, MIPF0000002). An additional CM was constructed using the bilaterian sequences from miRBase v.22, excluding both, paralogous sequences with 100% identity and sequences from the target species. All models were used as input for Infernal and for and Other_CM homology modes in miRNAture. All *let-7* retrieved sequences from miRBase were used as queries using BLAST mode in miRNAture with strategies 1,2,3, 5 and 6.

Both reference loci and the final results of miRNAture are stored in GFF3 format. Comparisons on genomic loci level were performed using bedtools [54] and classified as follows: *Match*: overlaps on the same strand; *Miss*: locus in references without overlap in miRNAture output; *Additional*: candidates detected by miRNAture without overlap in reference.

### 4.4. Curation of Human miRNA Families

miRNA precursor and mature sequences were retrieved from miRBase v.22 and corrected with MIRfix [19] to create a set of representative sequences for each miRNA family with a corrected set of mature positions, corrected precursor sequences and mature-anchored structural miRNA family alignment. From the latter family-specific covariance models where built using Infernal [22]. Specific command line parameters are described in Supplementary Section 6.

### 4.5. miRNA Annotation Using miRNAture

Covariance models where built for 350 miRNA families, excluding all sequences corresponding to the human genome from the dataset described in Section 4.4. A benchmark run of miRNAture was conducted to retrieve the final numbers from Section 2.3 where GFF3 files generated by miRNAture were compared to current annotation using bedtools v2.27.1 [54]. Specific command line parameters are described in Supplementary Section 6.

## Figures and Tables

**Table 1:** Re-annotation of the *let-7* family. For each species the number of loci annotated by miRNAture is shown at the homology stage (Homology) and after the evaluation stage (Final) and compared with the gold standard annotation merged from miRBase and Hertel et al. [40] (Annotation). We show how often the genomic coordinates from annotation match with candidate region (Match) or are not in the final candidate set (Miss) and the respective ratio over the total number of annotated regions. Candidates which pass the evaluation stage but do not overlap with annotation are counted as Additional. Labels: **Ann.**: Annotation, **Add.**: Additional.

Species	Homology	Final	MIRfix Filtered	Ann.	Match	Miss	Ratio Match	Ratio Miss	Add.
Human	26	20	6	14	13	1	0.928	0.07	7
Orang-Utan	27	18	9	14	13	1	0.928	0.07	5
Gorilla	26	20	6	14	13	1	0.928	0.07	7
Chimpanzee	30	24	6	14	13	1	0.928	0.07	11
Mouse	19	14	5	12	11	1	0.916	0.08	3
Sea squirt	7	6	1	5	5	0	1.0	0.0	1

**Table 2:** Comparison of Accepted/Filtered miRNAture miRNA candidates with respect to the current human miRNA annotation. For a final classification of miRNAture miRNA candidates, the latter are intersected with current miRBase v.22 annotation on genomic loci level. Candidates were classified as follows: **Accepted**: Candidate passed evaluation stage, **Filtered**: candidate did not pass evaluation. Numbers for all candidates of a specific family overlap (Perfect), some overlap (Partial) and no overlap (Without). Furthermore we investigate for how many families candidates currently not contained in the annotation of the corresponding family (Additional) are predicted or Filtered during evaluation. This set contains families from the Partial and Without class.

Class	Perfect	Partial	Without	Total	Additional
Accepted	284	28	11	323	178
Filtered	27	0	0	27	5

**Table 3:** Additionally predicted loci in comparison to current annotation for human (hg38). Reported numbers of families overlapping with each respective annotation category and the number of loci from this families in parenthesis. **d**: different miRNA, **r**: repeat and **o**: other non-intergenic region. Numbers were reported keeping a hierarchical comparison to avoid intersections between sets as: d>r>o.

	*d*	*r*	*o*	Total
Number	12 (13)	73 (685)	31 (245)	116 (943)
Fraction	0.010 (0.014)	0.629 (0.726)	0.267 (0.26)	

**Table 4:** Homology, structure and final filters applied on miRNAture. Specific programs used for each mode in parenthesis. *Ann.*: Annotation, *SS*: Secondary structure. *CSS*: Consensus secondary structure. ge: *gathering cutoff* from *Rfam* family. nBit=Bitscore/ge. ted: tree edit distance between default miRNA and modified multiple stockholm alignments. MFE: Minimum free energy. *HSPs*: high scoring pairs.

Sequence Homology		Alignment Score	Annotation/Structure Evaluation		Consensus Evaluation
Pairwise (blastn)	HMMs (nhmmer)	Alignment Score Evaluation (cmsearch)	Ann. Filter (MIRfix)	Secondary Structure (MIRfix)	SS Conservation
E-value ≤0.01	E-value ≤0.01	E-value ≤0.01	Ann. mature seq.	MFE <−10	ted≤7
≥20 nt HSPs			Seq. Length ≤200 nt		Valid CSS
Coverage ≥70%		Coverage ≥70%			
		nBit≥0.32∗ge $Biscore>\log_{2}2N$			

## Data Availability

Pre-calculated miRBase family alignments and corresponding CMs are available at http://www.bioinf.uni-leipzig.de/publications/supplements/21-001 (accessed on 31 January 2021). Data required to re-annotate human miRNAs including CMs and HMMs build from miRBase without human sequences are available at http://doi.org/10.5281/zenodo.4531376 (accessed on 31 January 2021).

## Supplementary Materials

The following are available online at https://www.mdpi.com/2073-4425/12/3/348/s1, Tabel S1: Blastn [2] strategies integrated in miRNAture for miRNAs detection on homology level. Strategies 1–4 are based on [6], strategy 5 on [3] and blastn default strategy (6); Table S2: Selection parameters dening representative sequences for the generation of miRNA CMs from miRBase . Parameters list as follows: *Target Clade* (target clade of available sequences); *Identity* (range of sequence identity between family members); Table S3: Strand-switch candidates detected by miRNAture in comparison to miRBase annotation for the human genome. **Overlap**: overlap state of predicted loci (either no overlap or partial); *: miRNAture predicts better match on opposite strand of miRBase annotation; Table S4: Additional loci on the strand opposite of human repeats.; Table S5: Listing of annotated human miRNA families with additional miRNAture predictions but no direct overlap due to ltering and reasons for that ltering in **bold**. Labels: **Ann**. Annotated, **Pred**. Predicted; Table S6: Human miRNAs annotated in miRBase where no miRNAture candidate passed the evaluation stage. General description of ltering in **bold**; Table S7: Overlaps between prediction of miRNAture with other annotated miRNA families from miRBase. Figure S1: Visualization of merging and annotation process performed by miRNAture to generate *extended regions*. blastn hits from strategy 1 are colored as brown tracks; Figure S2: Comparison of the intersection sizes between homology regions annotated by the available homology searches in miRNAture using blast, nhmmer (hmm) and Infernal searches in the human miRNA re-annotation; Figure S3: Discarded *let-7* sequences. **A**. *K-let-7* consensus structure derived from reported primate sequences in [3]. **B**. *G-let-7-1* locus from mouse; Figure S4: Annotation region for *mir-214*. miRNAture predict a previously unknown locus for *mir-214* thatoverlaps with sRNA-seq mapped reads (red regions) from [4]; Figure S5: New predicted *mir-548* locus in opposite strand overlap with a DNA repeat element from the family Tc1/Mariner (MADE1). Expression data (from [4]) is highlighted in red; Figure S6: Structural alignment of additional loci from *mir-544* family; Figure S7: A new predicted *mir-606* locus overlaps with a known lincRNA (lnc-GJA10-5). Additionally to the expression data from sRNA-seq (from [4] in red), the expression data of the lincRNA (from [1]) is highlighted as pale-blue tracks.

## Abbreviations

The following abbreviations are used in this manuscript:

miRNA	microRNA
ncRNA	non-coding RNA
CM	covariance model
HMM	Hidden Markov Models
