# Supplementary material for: miRNAture—Computational Detection of microRNA Candidates
Source: Genes (Basel). 2021 Feb 27;12(3):348. doi: 10.3390/genes12030348 (PMC7996739; doi:10.3390/genes12030348)
Supplement: Supplementary file 1 [file genes-12-00348-s001.pdf]

Supplement to: `miRNAture`: Computational detection of microRNA candidates

Cristian A. Velandia-Huerto, Jörg Fallmann and Peter F. Stadler

February 19, 2021

1 Processing steps to generate a `blast` hit's *extended region*

Figure 1 depicts the processing steps performed by `miRNAture` by the `blast` homology mode to generate *extended regions*. As an example, the detection of homologous candidates were performed on the scaffold `JH126831.1` from *Latimeria chalumnae* using the `blast` strategy 1. The raw mapped coordinates were labeled as `str1RawBlast` and comprise all the `blast` hits generated in this genomic region without any filtering. After refining the candidates a reduced number of hits remains (labeled as `str1FilteredBlast`). An additional iteration of merging was required in order to combine two or more filtered hits that overlap at their genomic coordinates. Comparisons were performed only in terms of genomic coordinates and their correspondent coverage with respect to queries (`str1FusionBlast`). The final product of those comparisons (the *region*) (`str1StrFinal`) was then subject to further evaluation with `cmsearch`.

2 `miRBase` covariance models for human miRNA families

`miRBase` v.22 human miRNA annotation and database files were downloaded via `FTP`. A cross-reference between `miRBase` accession number and miRNA family name was created via custom `Perl` scripts for later analysis of benchmark sets. 586 families representing a total of 1012 from 1913 currently annotated human miRNA *loci* could be curated. From this, a representative dataset of corrected multiple structural alignments was generated via `MIRfix` [7] and used to build family specific covariance models with `Infernal` [5]. For details about the combination of parameters to generate the final 350 CMs refer to Table 2. Command-line calls are listed in Section 6.

3 Discarded let-7 secondary structures from vertebrates

The structural evaluation performed in the final stage of `miRNAture` discarded annotated loci in primates (*K-let-7*) and mouse (*G-let-7*), based on their secondary structure depicted on Figure 3. In the first case, consensus structure for *K-let-7* sequences are reported, taking as reference the primate sequences from [3]. On second structure, `miRBase` (*mmu-let-7c-2*, `MI0000560`) let-7 sequence on mouse corresponded in [3] as a member of *G-let-7-1* paralogs.

4 Tables

Table 1: `Blastn` [2] strategies integrated in `miRNAture` for miRNAs detection on homology level. Strategies 1-4 are based on [6], strategy 5 on [3] and `blastn` default strategy (6).

**Blastn strategies**						
Flag	1	2	3	4	5	6
-dust	no					D
-soft_masking	false					D
-reward	5	4	5	4	D	D
-penalty	-4	-5	-4	-5	D	D
-gapopen	10	3	25	12	D	D
-gapextend	6	5	10	8	D	D
-word_size	7				D	D
-evalue	0.01				$10e^{-10}$	D
-outfmt	6					

Table 2: Selection parameters defining representative sequences for the generation of miRNA CMs from `miRBase`. Parameters list as follows: *Target Clade* (target clade of available sequences); *Identity* (range of sequence identity between family members).

**Strategy**		**Comments**	**Valid CMs**
Target Clade	*Identity*		
Mammalia	< 100	-	283
Vertebrata	< 100	Considering only high confidence sequences	12
Vertebrata	-	-	8
Vertebrata	$90 \leq 95$	-	3
Vertebrata	< 85	-	1
Mammalia	< 100	High confidence dataset	2
Primates	< 100		1
-	≤ 100	All available sequences were considered independent of confidence reported in `miRBase`, manual curation after iteration of parameters	34
Vertebrata	$90 \leq 100$	Manual curation after iteration of parameters	6
		Total	350

Table 3: Strand-switch candidates detected by `miRNAture` in comparison to `miRBase` annotation for the human genome. **Overlap**: overlap state of predicted loci (either no overlap or partial); *: `miRNAture` predicts better match on opposite strand of `miRBase` annotation.

**Overlap**	**Family**	**Annotation**	**Accession number**
None	mir-764 (MIPF0000707)	hsa-mir-764*	MI0003944
	mir-873 (MIPF0000390)	hsa-mir-873	MI0005564
	mir-140 (MIPF0000085)	hsa-mir-140*	MI0000456
	mir-1306 (MIPF0000531)	hsa-mir-1306*	MI0006443
Partial	mir-103 (MIPF0000024)	hsa-mir-103b-1*	MI0007261
		hsa-mir-103b-2*	MI0007262
	mir-101 (MIPF0000046)	hsa-mir-101-2	MI0000739
	mir-122 (MIPF0000095)	hsa-mir-122b	MI0017383
	mir-290 (MIPF0000068)	hsa-mir-371b*	MI0017393
	mir-451 (MIPF0000148)	hsa-mir-451b	MI0017360
	mir-515 (MIPF0000020)	hsa-mir-1283-2	MI0006430
	mir-548 (MIPF0000317)	hsa-mir-548aa-2	MI0016690
	mir-1245 (MIPF0000620)	hsa-mir-1245b	MI0017431
	mir-4536 (MIPF0001319)	hsa-mir-4536-1	MI0016906

Table 4: Additional loci on the strand opposite of human repeats.

**Family**	**Loci number**	**Repeat family**
mir-544	50	MER5A1
mir-548	42	MADE1
mir-1302	27	MER53
mir-1289	17,4	MER5A,MER5B
mir-649	19	MER8
mir-297	13	MER8
mir-1277	10	L1MC4, L1P5, LTR16
mir-574	9	Multiple short repeats: (TG)n or (GT)n
mir-483	9	GA-rich
mir-1285	7	Alu
mir-4536	5	L1MC4
mir-645	4	MER1A, MER1B
mir-559	4	Multiple families
mir-1262	4	Multiple families

Table 5: Listing of annotated human miRNA families with additional `miRNAture` predictions but no direct overlap due to filtering and reasons for that filtering in **bold**. Labels: **Ann.** Annotated, **Pred.** Predicted.

**Family**	**Ann. loci**	**Pred. loci**	**Accession**	**Comments**
mir-1233	2	21	hsa-mir-1233-1 (MI0006323) hsa-mir-1233-2 (MI0015973)	Family composed of 7 primate sequences, 2 from human. All mature sequences predicted by similarity to `MI0006323`. **Predicted mature sequence outside hairpin precursor.**
mir-1291	1	4	hsa-mir-1291 (MI0006353)	Family composed by 9 sequences, and multiple species. **Predicted hairpin precursor folds into invalid structure**.
miR-297	1	69	hsa-mir-297 (MI0005775)	Mouse specific validated family. Predicted miRNAs by similarity to primates. Human mature supported by 10 reads. **Annotated sequence too short for homology stage detection.**
mir-6127	1	1	hsa-mir-6127 (MI0021271)	Long secondary structure, experimental support only in human. Family composed of 3 sequences from chimp, macaque and human. **Predicted hairpin precursor folds into invalid structure**
mir-645	1	9	hsa-mir-645 (MI0003660)	Family composed of 3 primate sequences. Only one in human is supported by experiments. **Overlap between mir and mir* on predicted precursor.**
miR-652	1	11	hsa-mir-652 (MI0003667)	Predicted homology candidates on both strands. **Available annotated mature sequences did not match with predicted precursors. Low similarity.**
mir-877	1	1	hsa-mir-877 (MI0005561)	**Not possible to predict mir* based on available mature sequence located in loop or outside hairpin precursor.**
mir-873	1	1	hsa-mir-873 (MI0005564)	**Discarded annotated candidate due to folding into invalid structure.**
mir-1306	1	1	hsa-mir-1306 (MI0006443)	`miRNAture` **predicts loci opposite to annotation as valid.**
mir-140	1	1	hsa-mir-140 (MI0000456)	
mir-764	1	1	hsa-mir-764 (MI0003944)	

Table 6: Human miRNAs annotated in `miRBase` where no `miRNAture` candidate passed the evaluation stage. General description of filtering in **bold**.

**Family**	**Ann. loci**	**Accesion**	**Comments**
mir-1184	3	hsa-mir-1184-1 (MI0006277)<br>hsa-mir-1184-2 (MI0015971)<br>hsa-mir-1184-3 (MI0015972)	Family annotated only in human and chimpanzee. **More species should be annotated**.
mir-1207	1	hsa-mir-1207 (MI0006340)	Short predicted mature. **Invalid structure**.
mir-1224	1	hsa-mir-1224 (MI0003764)	Branched miRNA precursor. **Invalid structure**.
mir-1260b	1	hsa-mir-1260b (MI0014197)	Short predicted mature. **Broken mature prediction**.
mir-1282	1	hsa-mir-1282 (MI0006429)	Long sequence, mature sequences not matching. **Broken mature prediction**.
mir-1287	1	hsa-mir-1287 (MI0006349)	Structural evaluation failed for all members of this family. **Invalid structure**.
mir-1307	1	hsa-mir-1307 (MI0006444)	Short predicted mature. **Broken mature prediction**.
mir-1343	1	hsa-mir-1343 (MI0017320)	Mature sequence position does not fit annotation. **Broken mature prediction**.
mir-187	1	hsa-mir-187 (MI0000274)	Long and invalid structure. **Invalid structure**.
mir-331	1	hsa-mir-331 (MI0000812)	Mature did not match by one nt, predicted on both strands, alignment with gaps. **Broken mature prediction**.
mir-339	1	hsa-mir-339 (MI0000815)	Long and invalid structure.**Invalid structure**.
mir-384	1	hsa-mir-384 (MI0001145)	Annotated by similarity with mouse sequence (MI0001146). **Lack of experimental support**.
mir-454	1	hsa-mir-454 (MI0003820)	Mature did not match by 4nt, alignment with gaps. **Broken mature prediction**.
mir-484	1	hsa-mir-484 (MI0002468)	Not possible to locate mir* sequence in the predicted hairpin. **Broken mature prediction**.
mir-550	5	hsa-mir-550a-1 (MI0003600)<br>hsa-mir-550a-2 (MI0003601)	Predicted in both strans. **Failed evaluation**.
		hsa-mir-550a-3 (MI0003762)	Long and invalid structure. Predicted on both strands. **Invalid structure**.
		hsa-mir-550b-1 (MI0016686)	Predicted on both strands. **Failed evaluation**.
		hsa-mir-550b-2 (MI0016687)	Predicted on both strands. Located at same region as MI0016686. **Failed evaluation**.
mir-554	1	hsa-mir-554 (MI0003559)	Predicted mir* in loop and invalid structure. **Non-canonical mature**.
mir-593	1	hsa-mir-593 (MI0003605)	Not possible to predict mir*, overlaps mir and structure invalid. **Non-canonical mature**.
mir-601	1	hsa-mir-601 (MI0003614)	Invalid structure. **Invalid structure**.
mir-626	1	hsa-mir-626 (MI0003640)	Invalid structure. **Invalid structure**.
mir-631	1	hsa-mir-631 (MI0003645)	Not possible to predict mir*, overlaps mir and structure
mir-632	1	hsa-mir-632 (MI0003647)	invalid. **Non-canonical mature**.
mir-646	1	hsa-mir-646 (MI0003661)	Structural evaluation failed for all members of this family. **Invalid structure**.
mir-657	1	hsa-mir-657 (MI0003681)	Invalid structure. **Invalid structure**.
mir-665	1	hsa-mir-665 (MI0005563)	Not possible to predict mir*, overlaps mir and structure
mir-766	1	hsa-mir-766 (MI0003836)	invalid. **Non-canonical mature**.
mir-938	1	hsa-mir-938 (MI0005760)	Not possible to predict mature sequences. **Broken mature prediction**.
mir-940	1	hsa-mir-940 (MI0005762)	Not possible to predict mir*, overlaps mir and structure invalid. **Non-canonical mature**.

Table 7: Overlaps between prediction of `miRNAture` with other annotated miRNA families from `miRBase`.

**Predicted**	**Annotated**	**Region**	**Comments**
mir-633	MIR4679-1	10:89063332-89063416,+	Human specific family (hsa-mir-4679-1 and hsa-mir-4679-2).
mir-1271	MIR3169	13:61199802-61199874,-	Human specific locus (hsa-mir-3169).
mir-610	MIR5580	14:53948418-53948492,-	Human specific locus (hsa-mir-5580).
mir-423	MIR3184	17:30117082-30117162,-	Human specific locus (hsa-mir-3184).
mir-1295	MIR1295B	1:171101729-171101809,+	Human specific locus (hsa-mir-1295b).
mir-3151	MIR5008	1:227941597-227941674,-	Human specific locus (hsa-mir-5008).
mir-9	MIR4794	1:64579834-64579938,+	Human specific locus (hsa-mir-4794).
mir-499	MIR499B	20:34990397-34990473,-	Family not included in the `miRBase` family. Previously annotated as hsa-mir-499a.
mir-3173	MIR4773-2<br>MIR4773-1	2:151368335-151368408,-<br>2:151368336-151368411,+	Human specific family (hsa-mir-4773-1 and hsa-mir-4773-2).
mir-944	MIR5186	3:151565890-151565980,-	Human specific locus (hsa-mir-5186).
mir-616	MIR4471	8:100382757-100382853,+	Human specific locus (hsa-mir-4471).

5 Figures

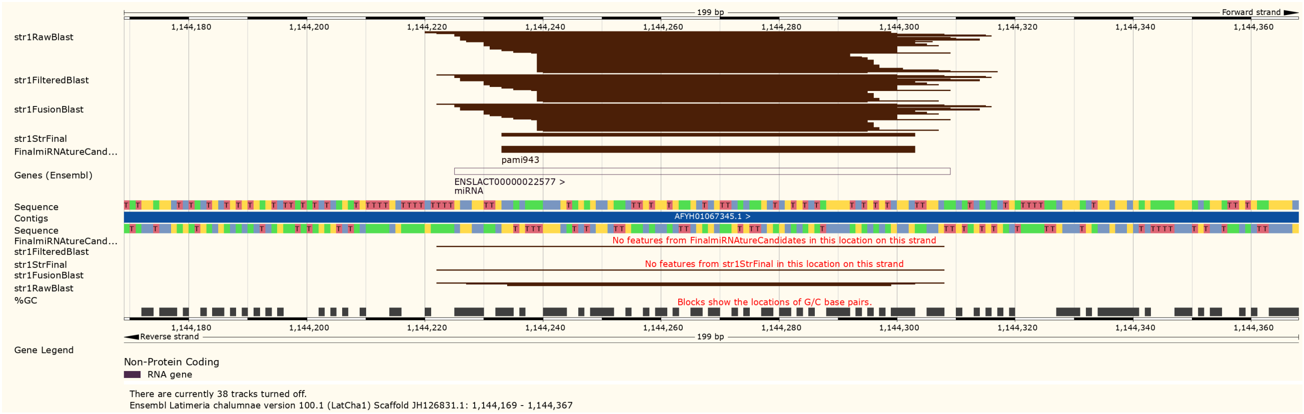

Figure 1: Visualization of merging and annotation process performed by `miRNAture` to generate *extended regions*. `blastn` hits from strategy 1 are colored as brown tracks.

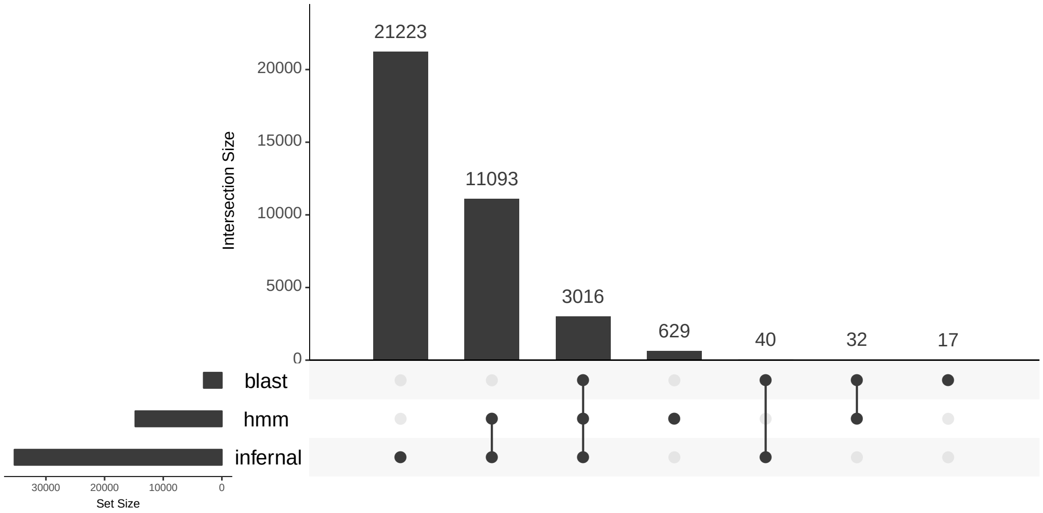

Figure 2: Comparison of the intersection sizes between homology regions annotated by the available homology searches in `miRNAture` using `blast`, `nhmmer` (hmm) and `Infernal` searches in the human miRNA re-annotation.

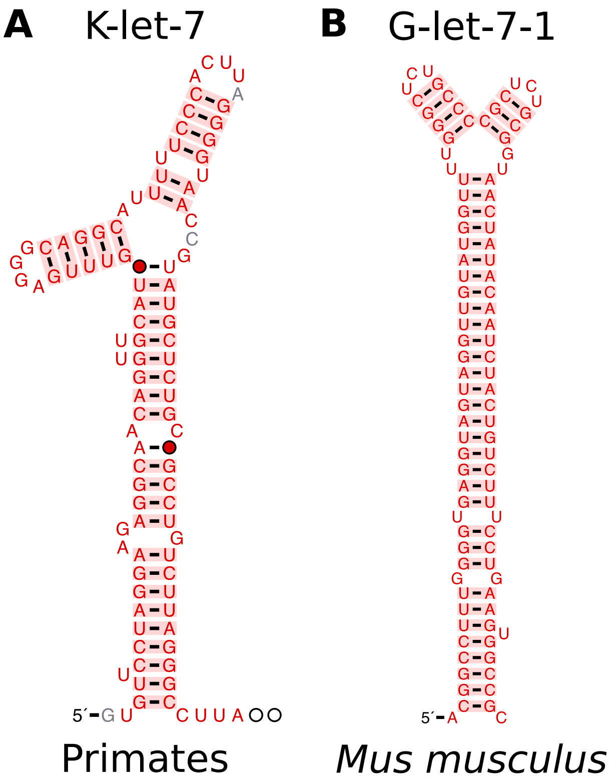

Figure 3: Discarded let-7 sequences. **A.** *K-let-7* consensus structure derived from reported primate sequences in [3]. **B.** *G-let-7-1* locus from mouse.

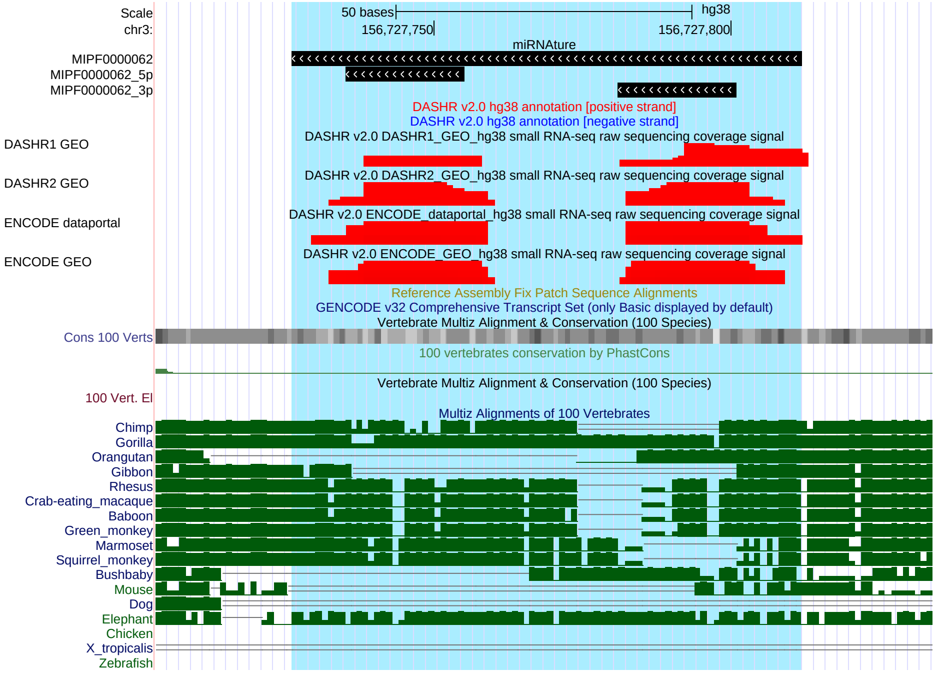

Figure 4: Annotation region for *mir-214*. `miRNAture` predict a previously unknown locus for *mir-214* that overlaps with sRNA-seq mapped reads (red regions) from [4].

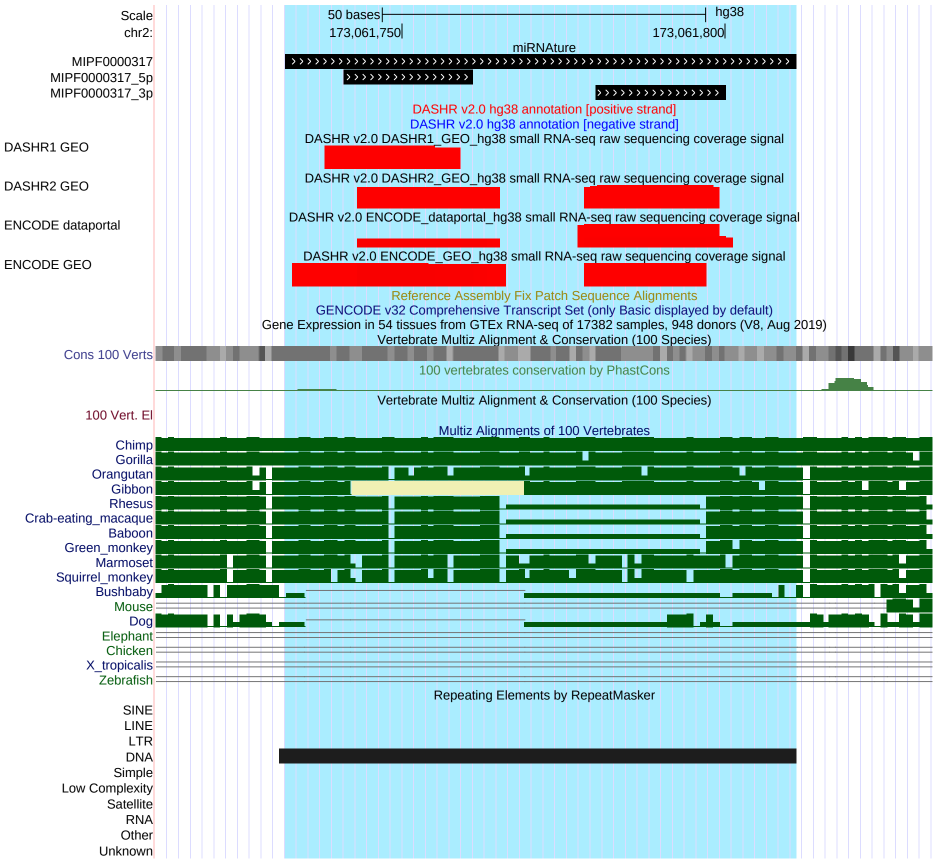

Figure 5: New predicted *mir-548* locus in opposite strand overlap with a DNA repeat element from the family Tc1/Mariner (`MADE1`). Expression data (from [4]) is highlighted in red.

```
# STOCKHOLM 1.0
H1609978131   -CAUUUG-GAGAUCUUGUUU--AAAAGCAGAUUCUAAUUCAG-UAGAUCUGACAUGAAGCCUAAGAGUC-UGCUUUUUUAACAAGCUCCCAAGUGCUG-
H1609978402   -CACCUG-GGCAUCUUGUUA--AAAUGCAGGUUCUAGUUCAG-UAAGGU------GGAGUCUGAAAUUC-UGCAUUUCUAACAAACUUCCAAGUGAUG-
H1609978495   -CACCUA-GGAAUUUUUU-AA-AAAUGCAGAUUCUGAUUA----AGGCCUGGGGUGGGGCCUGAGAUCC-UGCAUUUCUAACAAGCUUGCAGGUGAU--
H1609978520   -CACCCA-GGGAUCUUGUUA---AAUGCAGAUUCCGAUUCAG-UCAAUC-UGGAUGGAGCCUGAGAUUC-UACAUUUCUAACAAGCUCCCAGGUGAUA-
H1609978634   -CACCUG-GGGAUCUUAUUA--AAAUGUAUAUUUUAAUUCUG-UAAGUCUGGAUUGGGGCCUGAGAUUC-UGCAUUCCGAGUAAGCUCCCAAGUGCU--
H1609978674   -CACCUG-GGGAUCUUGUUAA--AAUGAAGAUUCUGAUUUAG-AGGUCAGA-GGUUGGG-CCA----------UUUU-UAACAAGCUCUCAGGUGAUG-
H1609979434   -UAUCUG-GGGAUCUUGUUA--AAAAGCAGAUUUUGGUUCAG-AAGUCU-AGGGUGGGGCCUGAGAUUC-UGUAUUUCUAACAAGCUCUCAGGUGAUU-
H1609979871   -CUCCUG-GGGAUCUUGUGA--AAAUGUAGAUUCCAAUUCAG-UUGGUC-UGAGUAAGGCCUGAGAGUC-UGCAUUUCCAACAAGCUCCUAGGUGAUG-
H1609979978   UCACCUG-GGGAUCUUGUUA--AAAUGCAGGCUCUAGUUCAG-GAGGUC-UGGAUGGGGCCUGAGAGUC-UGCAUUUCUAACAAGUUCCCAGUGAUGC
H1609980077   -CACUUA-GGAGUCUUGUUAA--AAUGCAGAUUCUGAGUUGG-UAAAUCUGAAAUAGGACCCGAGAUUC-UGCAUUUCUAACAAGCUCCCAGAUGAUG-
H1609980198   -UACCUG-GGGAUUUUGUUA--AAGUGAGGAUUCUG-------AAGGUCUGAGGUGAGACUUGAGAUUC-UGCAUUUCUAGCAAGCUCCCACGUGAU--
H1609980254   UUACCUG-GGGAACUUGUUAA--AAUGCAGAUUCUGA--CAG-UAGGUCUGGGGUGGGAUGUAUGAUUC-UGCAUUUCUAACAAGUUUCCAGGUGGUGG
H1609980357   -CACAUG-GGGAUCUUGUUA--AAAUUCAGAUUCUGAUUCACUUAGAUCUUGGAUGUGGCCUGAGAUUC-UGAAAUUAUAACAAGUUACCAGGUGAUG-
H1609980980   UCACCUG-GA-GUCUUAUUG--AAAUGAAGAUUCUGAUUCAA-UAGGCA-UGGGAUGGGCCUGAGAUUC-UGCAUUUCUAACAAGCUCUCAAGUGAU--
H1609981195   -CACCUG-GGAAUCUUGUUU--AAAUGCAGAUUCAGCUCAGG-UAGGUC--AGGUAGGACCUGAGAUUC-UGCAUUUCUAACAAGCUCUCAAGUGAU--
H1609981237   UCACUUA-GAGAUCUUGUCAA-AAAUGCAGAAUUUGAUUUAG-UAGGUCUGG-----GUCCUGAAAUUC-UGCUCUUCUAAUAAGCUCCCAAGUGAUGC
H1609981351   -CACCUG-GGGAUCUUGUUA--AAAUGUAGAUUCGAAUUCAG-UAAAUUUUGGAUGAAGCCUGAGUUUC-UGCAUUUCUAAUAUGCUCUCAUGUGAUA-
H1609981444   -CACUUU-AAGAUCUUGUUAA--AAUGCAGAUUCUGGUUAAG-CAGGAAUG------------AAUUC-UGCAUUUUAAACAAGCUCCCAGGUGAUU-
H1609981499   -CACCUG-GGGAUCUUGU-AA-AAAUGCAGAUUCUGAUUCAC-UAGUUUUGGGGUAGGGUCUGAGGGUC-UACAUUUCUAACAAGUUCUCAGGUGACU-
H1609981612   -GAUCUG-GGGAUCUUAUUA--AAAUGUAGAUUCUGAUCCAG-UCUGUCUGGAGUGGGGCCUGAGGUUC-UGCCUUUCUAGCAAGUUCCCAGGUGAUU-
H1609981678   --ACCUA-GGGAUCUUGUUA--AAAUGUAGAUUCUAAUUCAG-UGAGUC-CAGGGUGGGCCUGAGAUUC-UGAAUUUCUAGCAAGCUCUCAAGUGAU--
H1609981721   UCACCUG-GAGAUCUUGU-AA-AAAUGCAGAUUUUAAUUCAG-UGGGUCUCAGGUGGGGCCUGGGAUUC-UGCAUUUCUAACAAGUUC-CAGGUGAUG-
H1609982116   -CACCUG-GGGAUCCUGGAAAAAAAUGCAGAUUCUGAUUCAG-CAGGUCUGGGGUGCAGCCUGAGAUUC-UGCAUUUUUAACAAGCUCCCAGGUAAU--
H1609982321   GCACCCA-GGGAACUUGUUAA--AAUGCAGAUUCUGUUUCAG-CAAGUCUGAGAUGGAGCCUCAGAUUC-UGCAUUUCUAACAAGUUCCCAGAUGAUGC
H1609982944   UCACCUG--AGAAUUUGUUA--AAAUGCAGAUUCUGAUUCAG-UAUACCUGGAGUGGGGCCUGAAAUUC-UGCAUUUCUAAUAGACUCCCAGGUGAUG-
H1609982976   -CACCUG-GGGAUCUUGUUAA--AAUGCAGAUUUUGAUUCAG-UAUGUUUG-GGGUGGG-CCAGAGUUC-UACAUUU-UAACAAGCUCCCAUGUGAUG-
H1609983690   UCACCUG-GGGAUCUUGU-UA-AAAUGCAGACUUUGAUUCAC-AGGGGCUGGGGUGGGGUCUGAGGUUC-UGCAUUUCAAACAAUCUCCCAGGUGAA--
H1609983832   UUACCUG-GGGAGCUUGUUAA--AAUGCAGAUGCUGAUUCAG-GAAGUCUGA-----------GAUUC-UGCAUUUCUAUCAAGUUCUCAGGUGAUGC
H1609983881   UCACCUG-GGGAGCUUGUUA--AAAUGCAGAUUCUGAUUCUG-GAGUUC-UAGGGUGGGCCCAAGAAUC-UGUAUUUCUAGCAAACUCCCGGGUGAUGC
H1609984217   UCUCAUG-GAGAACUUGUUAA--AAUGCAGAUUCUCAUUCA---AUGUCUGGGGUGGGGCUUGAGAUUC-UGCAUGUGUAACAGGUUCCCAGGUGAUGC
H1609984397   -UAUCUG-GAAAUCUUGUUA--AAAUACAGAUUCUGAUUCAG-UAGAUU-GGGGUAGGGCCUGAGAUUC-UGCAUUUUUAUUAAGCCUCCAGGUGAUG-
H1609984484   -CACCUG-GGGAUUUUGUUAA--AAUGGGGAUUCUGAUACAG-UAAGGCCAAAAUGGAGCCUGGGAUUU-UACAUUUCUGACAAACUCCCAGGUGAUG-
H1609984569   -CAUCUA-GGGAUCUUGUUUA-AAAUGCAGGUUUUGAUUCUG-UUGACCCAGGGUGGGGCCUGGGAAUC-UGCAUUUCUAACAAGUUCCUAGGUGAUA-
H1609984936   -CACCUG-GGGAUCUUGUUA--AAAUGCAGAUUCUAAUUCAU-UAUUUC-UG----GGGCUUGAGAAUC-UGCAUUUCUAACAAGUUCCCAGGUAAUG-
H1609985061   --ACCUGGGGGAUUUUGUUA--AAAUGCAGAUUCUGAG-----UAGUUCUAG-GUGGGACUUGUGAUUCUUGCAUUUUUAACAAGCUCCCAGGUGA---
H1609985346   -CUUCUG-GACAUCUUGUUG--AAAUGCAGAUUCUAAUUCAG-UAAGUCUGGAAGAGGGUCUGAGAGUC-UGCAUUUCUAACAAGCUUCCAAGAGAUG-
H1609985469   --ACCUA-AGGAUCUUGUUU--AAAUGCAGAUUCUGAAUCAG-UAGGUCUGGGGUGAGGCCUGAGAUUC-UGUAUAUUUAGCAAGCUCCCAGGUGAU--
H1609985476   UCACCUG-GGAACCUUGUUA--AAAUGCAGAUUUUGAUUCAG-UAGCUCUGGCCCGGGGCCUGAGAGUC-UGCGUUUUUAGCCAGUUUCAAGUGCUG-
H1609985498   -CACCUG-GAAAUCUUGU-UA-AAAUGCAGCUUUCAACUUGG-CAGCUCUGGGGCAGGGCCUGAGAUUC-UGCUUUUCUAACAAGCUUCCAGGUGGU--
H1609985588   --UCCUG-GGGAUCUUGUUA--AAAUGCAGGUUGCAAUUCAG-AAGGUC-UGGGCUGGGCCUGAGAUUC-UGCAUUUCCAACAAGCUCUCAGGUGAU--
H1609985941   -CAUCUG-GACAUCUUGUUA--AAAUGCGGAUUCUGGGUCUG-UAGGUCUGGGGUGGUGCCUGAGAGUC-UGCGUUUCCAGCAAGCUCUCAGGUGAUG-
H1609986070   UCACCUG-GGGACCUUGUUAG--AAUGCAGAUUCUGAUUCAG-CAAGUCAG-CUUGGGGCCUGGGGUUC-UGCAUUU-UAACAAGCUCCCAGGUGAUUC
H1609986088   --ACCUAGAGGAUCUUUUUG--AAAUGCAGUUUCUGAUUCAG-UACAUCUGGAGUGAAGCCUGAGAU---UGCAUUUCUAGCAAGCUCCCAGGUGAUG-
H1609986585   -CACCUG-AUGAUCUUGUUAA--AAAGAAGAUACUGAUUCAG-UAGGUCUGA-----------UAUUC-UGCAUUUCCACCUAGCUCUCAGGUGAUG-
H1609986683   UCACCUG-GAAAUCUUGUUA--AAAUGCAGAAUCUAAUGGGG----------GUGGGAUGCUGAGAUUC-UACAUUUCUAAAAAGCUACCAGGUGAUGC
H1609986899   -CACCUG-GGAAUCUUGC-AG-AAAUGCAGAUUCUGACUCAG-CAGGUCUGACCUAGGGCCUGGGGUUC-UGCAUUUUUAACAAGCUCCCAGGUGAU--
H1609987364   UCACCUG-GGGAGCUUGUUAA--AAUGCAGGCUCUGUUUCU---GGGUCCCGGGUAGGGCUCUAGAUUC-UGCAUCUCUAACAAGUUCUCAAGUGAUGC
H1609987432   -UACCUG-GGGAUUUUGC-UG-AAAUGCAAA-ACUGAUUCAG-AAGGCUGGGGAUAAGGCCAGGGAUUC-UGCAUUUCUAACAAGCUCCCAGGUAAU--
H1609987696   -CACCUA-GAGAUCUUGUUAA--AAUGCAGAUUCUGACUUAA-UAGAUCUGGGAUAGAGACUGAAAUUC-UGCCUUUCUAGCAUGCUCUCAGAUGAUA-
H1609987842   --ACUUA-GGGAUCUCUUUAA--AGGGCAGAUUCUGAUUCCC-UAGACCUGA-----------GAGUC-UGCAUUUCUAACAAGCUCCCAGGUGAU--
#=GC RF       _CACCUG_GGGAUCUUGUUA__AAAUGCAGAUUCUGAUUCAG_UAGGUCUGGGGUGGGGCCUGAGAUUC_UGCAUUUCUAACAAGCUCCCAGGUGAUG_
#=GC SS_cons  .((((((.(((..((((((....((((((((.(((...........................))).)).))))))...)))))).)))))))))....
//
```

Figure 6: Structural alignment of additional loci from *mir-544* family.

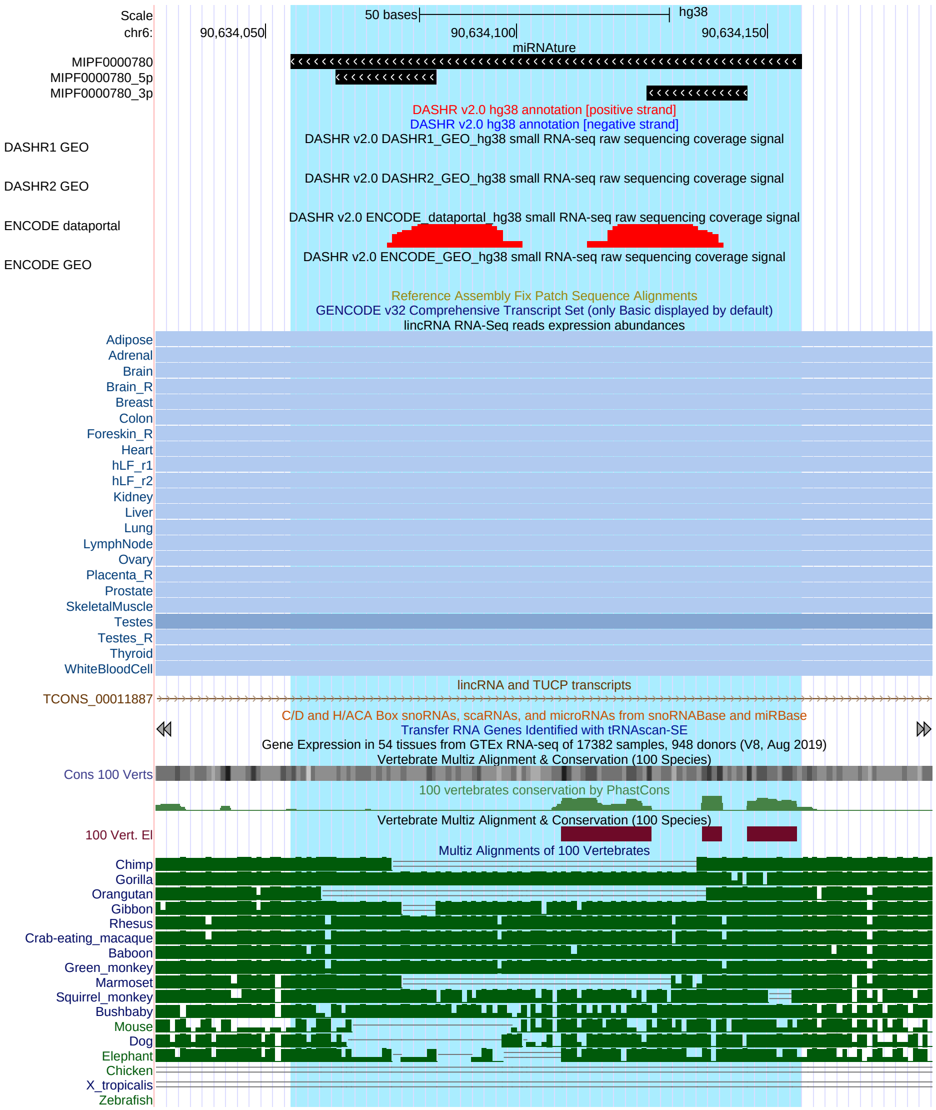

Figure 7: A new predicted *mir-606* locus overlaps with a known lincRNA (lnc-GJA10-5). Additionally to the expression data from sRNA-seq (from [4] in red), the expression data of the lincRNA (from [1]) is highlighted as pale-blue tracks.

6 Command line Methods

```
./miRNAture -stage complete -sublist <LIST Let-7 CMs> -speG <Path_specie_genome> \\
    -speN <Specie-name> -speT <Specie-Tag> -w <OUTPUT_DIR> -mfx <Path_MIRFix> \\
    -m <Blast,HMM,OTHER_CM,Infernal,Final> -pe 1 -str <1,2,3,5,6,ALL> -blastq \\
    <Blast_queries_folder> -rep default,150,100
```

Listing 1: `miRNAture` parameters to annotate let-7 loci on vertebrate genomes.

```
./miRNAture -stage complete -sublist <LIST HUMAN CM> -speG <Human Genome> \\
-speN "Homo sapiens" -speT Hosa -w <OUT FOLDER> -mfx <Path_MIRFix> \\
-m <Blast,HMM,OTHER_CM,Final> -pe 1 -str <5,6,ALL> \\
-blastq <Blast_queries_folder> -rep default,150,100
```

Listing 2: Re-annotation of miRNAs on human genome.

```
# Clustal omega
clustalo -i <multifasta file> --outfmt clu -o <output file>
# ViennaRNA packgage
RNAalifold --aln-stk=<fasta file> <align file>
# INFERNAL package
cmbuild <Covariance Model> <STO file>
cmcalibrate --cpu=20 <CM>
cmsearch --cpu 4 --tblout <TABULAR OUT> -o <OUT> <CM> <GENOME>
```

Listing 3: Modification of Covariance Models

References

[1] Moran N. Cabili, Cole Trapnell, Loyal Goff, Magdalena Koziol, Barbara Tazon-Vega, Aviv Regev, and John L. Rinn. Integrative annotation of human large intergenic noncoding rnas reveals global properties and specific subclasses. *Genes & Development*, 25(18):1915–1927, 2011.

[2] Christiam Camacho, George Coulouris, Vahram Avagyan, Ning Ma, Jason Papadopoulos, Kevin Bealer, and Thomas L. Madden. BLAST+: architecture and applications. *BMC Bioinformatics*, 10:421, 2009.

[3] Jana Hertel, Sebastian Bartschat, Axel Wintsche, Christian Otto, The Students of the Bioinformatics Computer Lab 2011, and Peter F. Stadler. Evolution of the let-7 microRNA family. *RNA Biology*, 9:231–241, 2012.

[4] Pavel P Kuksa, Alexandre Amlie-Wolf, Živadin Katanić, Otto Valladares, Li-San Wang, and Yuk Yee Leung. DASHR 2.0: integrated database of human small non-coding RNA genes and mature products. *Bioinformatics*, 35:1033–1039, 2018.

[5] Eric P. Nawrocki and Sean R. Eddy. Infernal 1.1: 100-fold faster rna homology searches. *Bioinformatics*, 29:2933–2935, 2013.

[6] Cristian A Velandia-Huerto, Adriaan Gittenberger, Federico D Brown, Peter F. Stadler, and Clara I Bermúdez-Santana. Automated detection of ncRNAs in the draft genome sequence of a basal chordate: The carpet sea squirt *Didemnum vexillum*. *BMC Genomics*, 17:591, 2016.

[7] Ali M. Yazbeck, Peter F. Stadler, Kifah Tout, and Jörg Fallmann. Automatic curation of large comparative animal microRNA data sets. *Bioinformatics*, 35:4553–4559, 2019.
